# A Compact Ultra-Wideband Monocone Antenna with Folded Shorting Wires for Vehicle-to-Everything (V2X) Applications

**DOI:** 10.3390/s23136086

**Published:** 2023-07-01

**Authors:** Martin Wooseop Lee, Feras Abushakra, Zachary Choffin, Sangkil Kim, Hee-Jo Lee, Nathan Jeong

**Affiliations:** 1 Electrical and Computer Engineering Department, The University of Alabama, Tuscaloosa, AL 35487, USA; wlee49@crimson.ua.edu (M.W.L.); zmchoffin@crimson.ua.edu (Z.C.); 2 Department of Electronics Engineering, Pusan National University, Busan 46241, Republic of Korea; ksangkil3@pusan.ac.kr; 3 Department of Physics Education, Daegu University, Gyeongsan 38453, Republic of Korea; hjlee@daegu.ac.kr

**Keywords:** vehicle to everything, low-profile antenna, ultra-wideband

## Abstract

In this paper, a capacitively-fed, ultra-wideband (UWB), and low-profile monocone antenna is proposed for vehicle-to-everything (V2X) applications. The proposed antenna consists of a monocone design with an inner set of vias. Additionally, an outer ring is added with a small gap from the monocone and shorted with six folded wires of different lengths to extend the operating band. The proposed antenna covers the frequency range from 0.75 GHz to 7.6 GHz and has a 164% fractional bandwidth, with a gain value varying between 2 and 10 dBi. The dimensions of the antenna are 0.37$\lambda_{L}\;$× 0.37$\lambda_{L}\;$× 0.067$\lambda_{L}$. The antenna was fabricated using a 3D printer with low-cost polylactic acid plastic (PLA) material and then sprayed with aerosol copper nanoparticles. The efficiency was approximately 90% throughout the frequency bands of interest. Finally, the proposed antenna was installed on a vehicle and tested with an OBU (onboard unit) and a RSU (roadside unit) in the field. The results show a longer wireless communication range for V2X applications.

## 1. Introduction

Vehicle-to-everything (V2X) technology is gaining increased interest in modern automotive engineering. Vehicle roof-mounted antennas are used for a wide range of applications such as vehicle-to-vehicle (V2V), vehicle-to-infrastructure (V2I), vehicle-to-network (V2N), vehicle-to-pedestrian (V2P), and keyless entry [1]. There are mainly two V2X technologies. One is DSRC (dedicated short-range communication) defined in IEEE 802.11c and the other is C-V2X (cellular-V2X) specified in 3GPP (Third-Generation Partnership Project). Both technologies operate at the band of 5.9 GHz. However, a modern vehicle wireless communication system is required to support other bands for road assistance applications, e.g., cellular communication for emergencies, Bluetooth, and Wi-Fi hot spots to create a local wireless network, and navigation technology [2]. Therefore, a modern vehicle wireless communication system must cover a wide frequency range to enhance road safety and transportation efficiency. To enable communication between a vehicle and the various wireless technology elements, the antenna needs to support the entire V2X band and beyond with vertical polarization and an omnidirectional radiation pattern. Since vehicles have multiple communication methods, excluding future V2X applications, a wideband antenna would help to minimize the number of antennas that need to be installed for each communication method. In addition, a low profile and structure fidelity are preferred features when mounted on a vehicle’s roof. Various antenna types can be used to fulfill the aforementioned requirements. Among different radiators, the monocone antenna, as a monocone-shaped or metal patch [3], is a well-known radiator that is widely used for vehicle applications. A conventional monocone antenna operates in TEM mode [4]. However, by adding shorting pins to the antenna, the TEM wave cannot propagate at the lower frequency band where the antenna height is shorter than a quarter wavelength, while a set of TM or quasi-TM modes can propagate [5,6]. These modes are vertically polarized modes with omnidirectional radiation patterns. Many methods have been proposed to reduce monocone antennas’ height and improve impedance matching. For example, dielectric loading was investigated in [6] to improve monocone antenna impedance matching. Loading a dielectric substrate near the feed region has been shown to improve the impedance matching of the monocone antenna and results in a greater reduction in monocone height [7]. Furthermore, miniaturizing the monocone antenna by applying a high permittivity dielectric hemisphere that completely encloses the conical radiator above the ground plane is shown in [8]. It shows that the high dielectric constant material is applicable to different dielectric materials with different permittivity values. Also, using grounding vias or a shorted-capacitive cap can improve impedance matching at the lower frequency band without the need to increase the size [9,10,11,12,13]. However, there is a trade-off between the number of shorting pins and the matching improvement over the entire operating band. Thus, the number of shorting vias and their locations need to be optimized carefully for optimum performance. 

In a similar approach, identical printed shorting strips on a dielectric substrate were utilized between the top hat and the ground [14,15,16,17]. However, it had the drawback of increasing the weight and air resistance once the vehicle accelerates. In addition, creating slots in the outer sides of the patch that surrounds the cone showed significant effects on the antenna bandwidth and achieved a 97% fractional bandwidth [18]. Another approach by adding a top hat with different shapes to the cone is reported to achieve better matching between the antenna and the feeder at the lower band [19,20,21,22]. A lightweight monocone antenna integrated into a hollow cavity is also reported and showed the trade-off between the cavity size and the antenna [23]. In addition, monocone antennas can be designed with end-fire radiation patterns. The radiation pattern of the end-fire monocone is improved either by adding grounded cones near the main cone to serve as reflectors [24] or by inserting a direct-fed dual loop with a horizontal current [25]. A dual-monocone antenna design is proposed to widen the bandwidth up to 185% by exciting the upper monocone with the feeding structures that are located inside the lower monocone [26].

Recently, 3D-printed antenna designs have gained more interest due to the recent advancement in 3D printing technology and the reduction in its costs [27,28]. Such technology offers rapid production with a relatively low cost and gives an interesting alternative to traditional 2D antennas.

In this paper, a low-profile capacitively-fed monocone antenna is presented to support V2X applications. The proposed design covers the frequency band between 0.75 GHz to 7.6 GHz, with a 164% impedance bandwidth while keeping a compact size and low profile. The design is realized by adding two sets of grounding configurations that are utilized to achieve significant miniaturization. The inner set is four straight conducting posts between the monocone and the ground, while the outer set is six folded shorting wires to extend the bandwidth. The folded wires have different lengths to further extend the operating band at the lower frequency end. The position and angular spacing between the shorting posts were chosen to improve impedance matching and are presented in this paper. The antenna is fabricated with a 3D printer before the copper coating. The radiation pattern of the proposed design is stable over the frequency of interest. A field test of the proposed antenna was utilized to validate its performance and compare it with a commercially available antenna used for the same applications.

## 2. Antenna Design and Geometry

**Figure 1:**
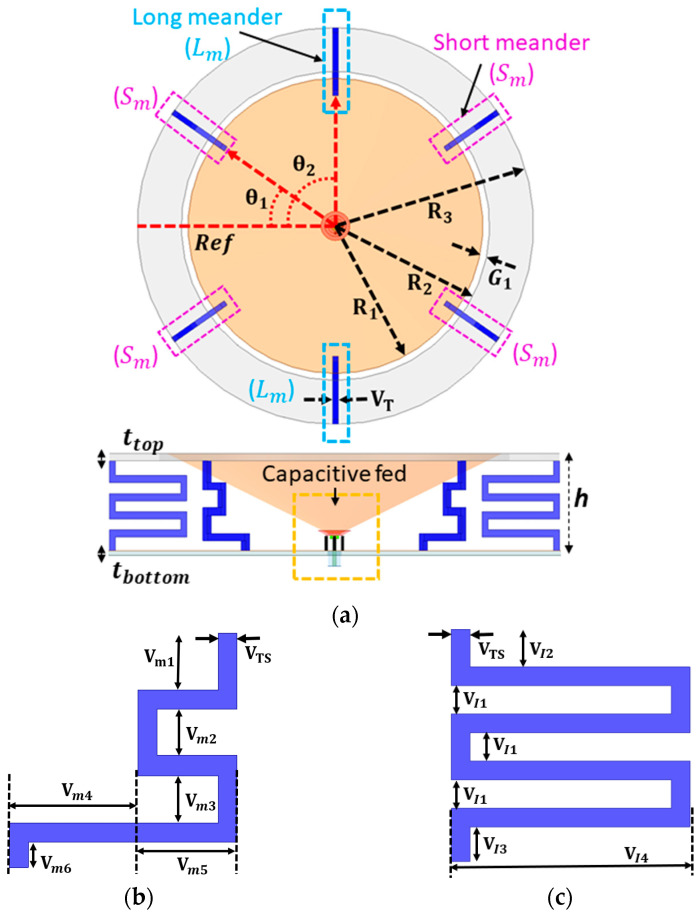
Proposed monocone antenna: (**a**) top and side view, (**b**) short folded meander lines, and (**c**) long folded meander lines.

The geometry of the proposed antenna is shown in Figure 1. The antenna design contains five components—a monocone, a capacitive feeder, inner grounding posts, a grounding ring, and folded shorting lines. The tapered monocone is introduced to provide a broad bandwidth by optimizing its height (h) and top monocone radius (R_1_). A capacitive feed is incorporated to allow broad impedance matching over the frequency band of interest. The height and upper radius of the feed are defined as RC_2_ and RC_1_, respectively. The design is placed on a metal plate with a thickness of (t_bottom_ = 1.5 mm). The gap (G_2_) between the monocone and capacitive feeder is filled with a thin-film dielectric with a relative permittivity (ε_r_) of 2.02 and a loss tangent (tan δ) of 0.0002. Figure 1b,c illustrates the geometry and design of the proposed folded shorting meander lines. 

**Figure 2:**
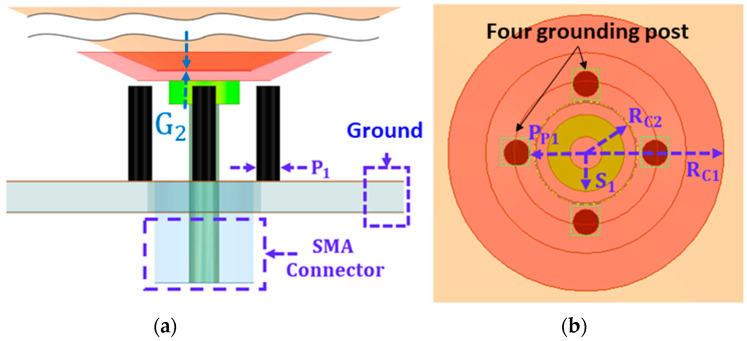
Capacitive feed structure: (**a**) side and (**b**) top view.

Figure 2 includes the geometry and dimensions of the capacitive feed. A tapered V-shape conductive feed is designed and positioned at the bottom of the monocone to provide electric coupling from the 50-ohm coaxial feed to the monocone. The center and outer conductors of the SMA connector are physically touched by the capacitive feeder and ground plane. The overall height and diameter of the proposed antenna are 26.7 mm and 148 mm, respectively. The grounded cylinder post near the capacitive feed has a width of P_1_. 

A total of four inner grounding posts are located 90° apart from each other. The posts were installed near the capacitive feed to improve impedance matching at the lower frequency range. A planar outer ring is placed beside the open-ended edge of the monocone with a small gap (G_1_) and is grounded through six folded shorting lines. 

These lines are vertically located and connect the ground ring to the ground plane. Two of these folded lines are the long meander (L_m_), while the other four are the short meander (S_m_). The folded wires are 2 mm-thick. The thickness of the meander line was also considered for better rigidity to properly support the ground ring while optimizing low band matching performance. 

For the short meander, the V_m4_ length was also optimized to control the amount of capacitive coupling with the electrical length. A long meander line is added to extend the electrical length to expand the coverage of the low-frequency band with an optimized short meander. Both long and short meander lines have different-sized gaps between the lines to achieve better optimization for the low-frequency band. A combination of both long and short meanders was able to result in better coverage for low-band antenna performance. The angles (θ_1_) of the four short meander lines (S_m_) are located at 35°, 125°, 195°, and 325° from the reference line. The angles (θ2) of the two long folded lines are located at 90° and 270°. The spacing between the monocone and the ring was adjusted to control for the amount of capacitive coupling along the perimeter of the monocone. The inner and outer radii of the ring are denoted as R_2_ and R_3_, respectively. Table 1 includes the parameters and dimensions of the proposed capacitive-fed antenna which are shown in Figure 1 and Figure 2.

## 3. Results and Discussion

**Figure 3:**
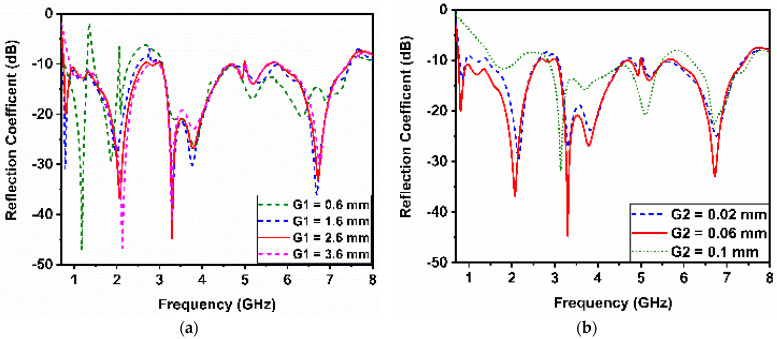
Effects of: (**a**) variation in G_1_ and (**b**) variation in G_2_.

The effect of the spacing between the open-ended edge of the monocone and the grounded ring is examined by varying G_1_ from 0.6 mm to 3.6 mm. The result indicates that in the lower frequency band regions, the spacing needs to be approximately more than 2.6 mm to avoid frequency notches in the lower frequency band. This is mainly because of the rapid changes in the input impedance imaginary part of the design with the open-edge distance variation. The separation distance (G_2_) between the monocone and capacitive feed varied from 0.02 mm to 0.1 mm. The simulated results show that if the gap increases to 0.1 mm, the reflection coefficient significantly degrades across the frequency range of 700 MHz to 3 GHz. As separation decreases to 0.02 mm, the return loss across the same region improves, as shown in Figure 3. This is because the capacitive feeder compensates for the inductive characteristic of the monocone. 

**Figure 4:**
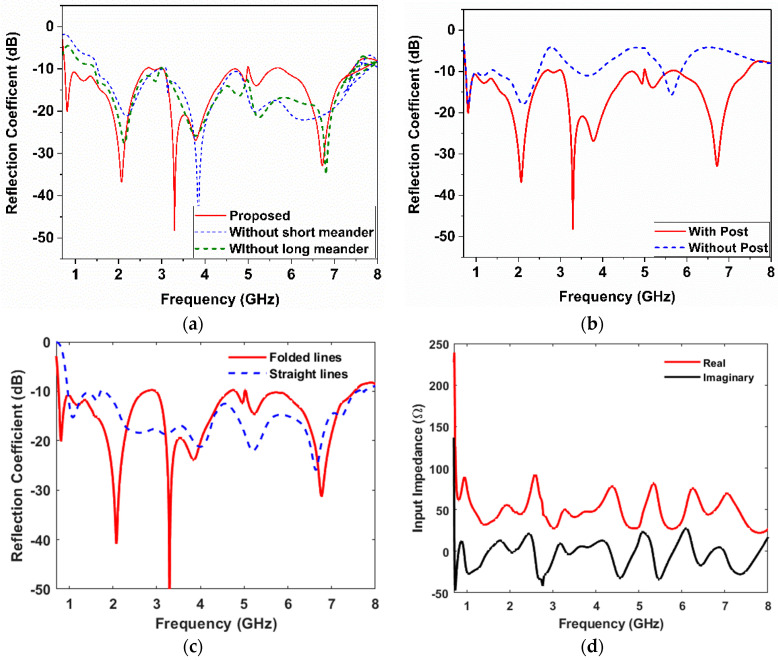
Reflection coefficient: (**a**) without long/short meanders, (**b**) with and without posts, (**c**) with the proposed folded and straight lines, and (**d**) the input impedance with meanders and posts.

The impact of the folded wires near the capacitive feed is also investigated. Significant improvement was observed in the presence of the ground posts throughout the frequency band of interest, as illustrated in Figure 4a. It could be seen that both long and short folded wires improve matching at the same frequency range. However, when both of them were incorporated into the design, the bandwidth was extended further toward the lower frequency range. Figure 4b illustrates the overall effects of the grounding posts on the reflection coefficient of the design. It is clear that these grounding posts greatly improve impedance matching throughout the entire frequency range of interest. In addition, to compare the effect of the proposed folded wires relative to the traditional straight shorting lines, the reflection coefficient of both cases is plotted in Figure 4c. It is clear that the folded wire structure results in more miniaturization relative to the straight-line shorting pins that have been widely investigated in the literature. The folded meanders shifted the operating frequency to 750 MHz, while the straight lines achieved a low operating frequency band starting at 1 GHz. The flexibility in matching a UWB antenna with this design approach is considered interesting as it can be applied to many other antenna types. Figure 4d shows the input impedance of the final design stage where the real part is around 50 Ω and the imaginary part is around 0 Ω.

**Figure 5:**
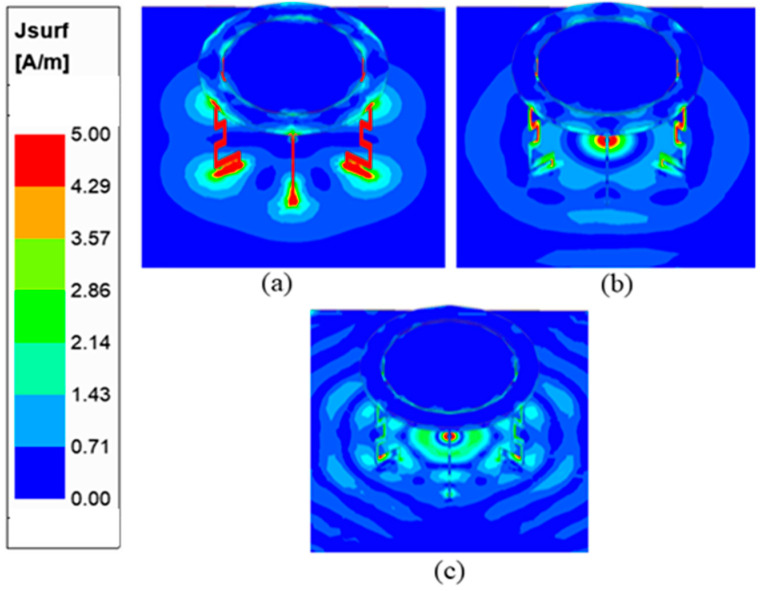
Current distribution for (**a**) 0.77 GHz, (**b**) 3.5 GHz, and (**c**) 5.9 GHz.

These folded shorting wires in the proposed design result in the miniaturization of the antenna by shifting the lower frequency band by 250 MHz. To identify the radiating part of the proposed antenna, the current distribution is shown at low, middle, and high frequencies, as illustrated in Figure 5. The magnitude of the surface current at 0.77 GHz is concentrated on both the long and short meander lines. Since there is significant reflection coefficient variation when either of the meander lines is removed, the current distribution shows that the short and long meander lines affect the low-frequency regions near 0.77 GHz. The proposed antenna operates in the quasi-TM modes similar to [6]. The surface current in the 3.5 GHz region is concentrated on the capacitive feed and meander lines.

## 4. Antenna Fabrication and Measurements

**Figure 6:**
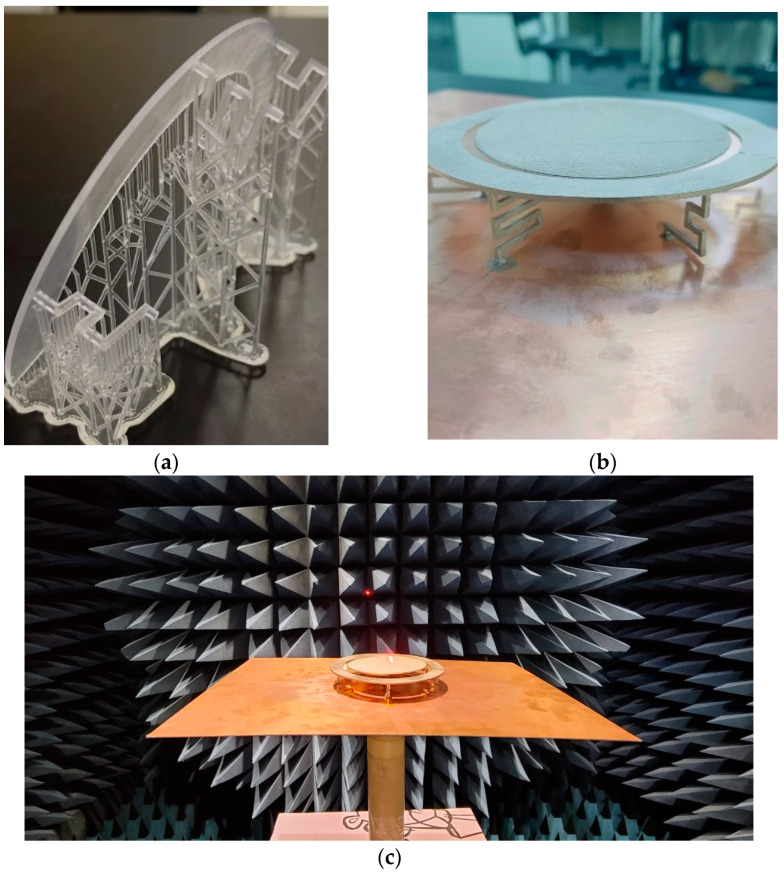
Fabricated antenna: (**a**) before the copper coating, (**b**) after the copper coating, and (**c**) the antenna test in the chamber.

All components of the proposed antenna are made from low-cost and rigid polylactic acid plastic (PLA) material, with dielectric losses of 0.01, and resin. A 3D printer (Dramel 3D45) and a resin printer (Formlabs Form 3) were used to precisely fabricate the antenna geometry. To make the antenna lightweight, cost-effective, and electrically conductive, MG Chemical’s Super Shield copper conductive nanoparticles (843AR-140G) were utilized for metallization and sprayed on the components followed by drying for 40 min without any directly blown air. The conductive spray was applied only once to cover all of the PLA material of the antenna. The small variation in the copper thickness, 1~2 oz., did not show a significant effect on the antenna’s performance [29,30]. The conductivity of the copper particle is 3300 S/cm, which is almost 200 times less conductive relative to the industrial copper-clad laminate of 1 oz. For the reduction of weight and costs, while keeping structural fidelity, the monocone structure of the antenna has an infill ratio of 80%. Figure 6 depicts the fabricated antenna component before and after copper-particle coating. The electrical connection of the shorting posts to the ground plane is also realized with the same spraying method. This simple metallization is desirable for rapid mass production and cost reduction.

**Figure 7:**
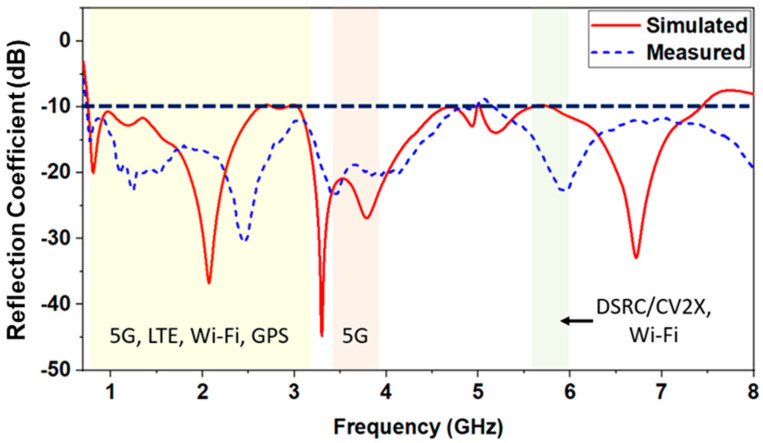
Simulated and measured reflection coefficient.

The simulated and measured reflection coefficients of the proposed antenna are shown in Figure 7. The simulation results indicate that the antenna supports an ultra-wide bandwidth covering 0.75 GHz to 7.6 GHz, with −10 dB of the reflection coefficient criteria. Some differences between the simulated and measured reflection coefficients are due to fabrication tolerance. However, they are still in good agreement. The operating band covers GSM800, GSM900, GPS, DCS/GSM1800, PCS/GSM1900, WCDMA/UMTS/IMS-2000, WiBro, LTE Band 1/2/4/7/13/17, WLAN, and DSRC/C-V2X.

**Figure 8:**
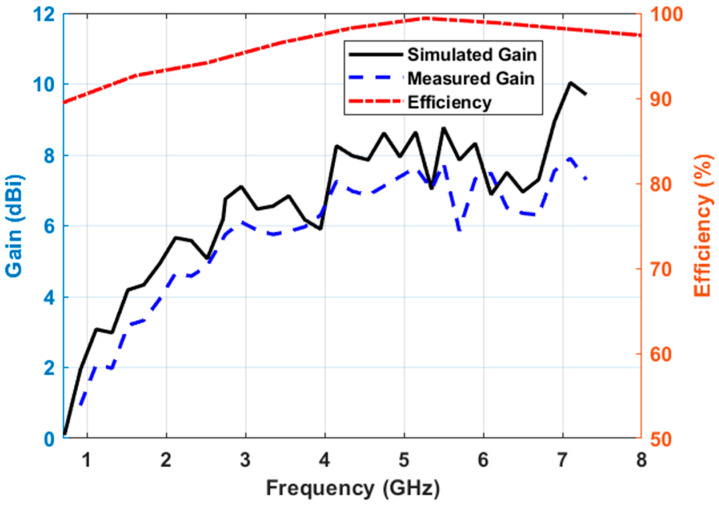
Peak realized gain and efficiency.

The proposed antenna was tested to validate the far-field characteristics of the antenna in the Howland Model 3500D anechoic chamber. The measured peak realized gain varied between 2 to 10 dBi at the frequency band of interest, as shown in Figure 8. The gain at the center frequency is approimately 7 dBi. The gain value is comparable to the theoretical gain value from the radiation aperture area. The antenna efficiency is above 90% throughout the operating band.

**Figure 9:**
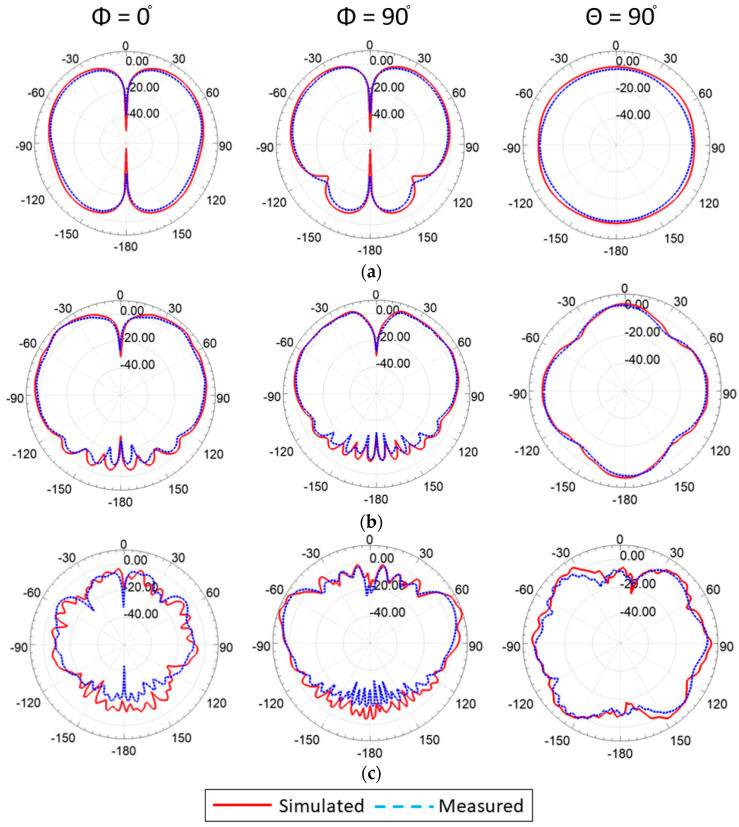
Simulated and measured radiation patterns of the proposed antenna: (**a**) 0.77 GHz, (**b**) 3.5 GHz, and (**c**) 5.9 GHz.

**Figure 10:**
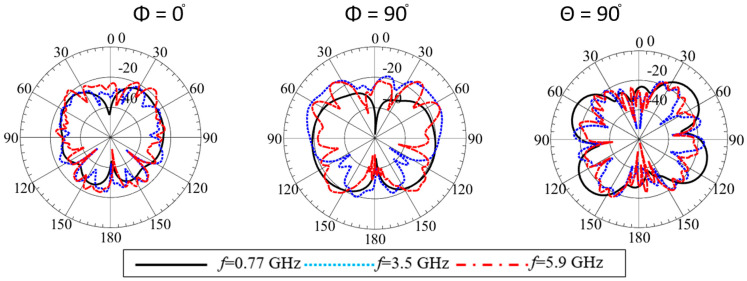
Simulated cross-pol radiation patterns of the proposed antenna at different frequencies.

The radiation patterns were plotted in two principal planes at the representative frequencies, as shown in Figure 9. The dipole-like patterns were observed at the different frequencies near the angular range of θ = ±90°, which is required to communicate to nearby traffic infrastructure and cellular towers. In addition, at θ = 90°, the omnidirectional patterns were measured between φ = 0° to φ = 360°, ensuring full horizontal coverage to wireless connection for nearby vehicles and the power grid infrastructure. Furthermore, the cross-polarization level was found to be less than −25 dB at the lower frequency band and less than −20 dB at the higher frequency end, which is expected due to the higher-order modes’ propagation, as shown in Figure 10. The simulated and measured radiation patterns are in good agreement.

## 5. V2X Field Test Results

**Figure 11:**
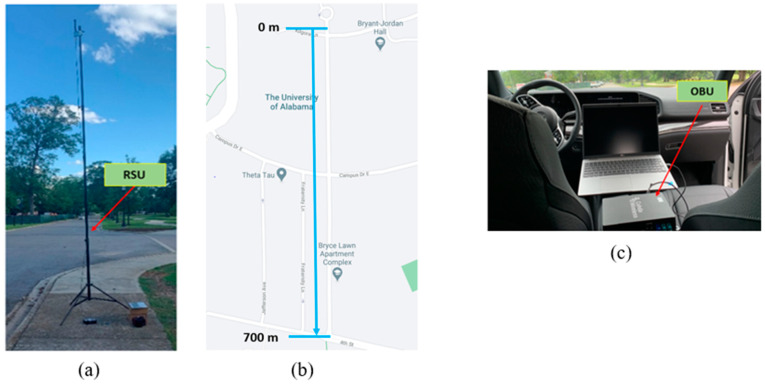
Field test setup: (**a**) the RSU with a monopole antenna at a 17 foot-high mast, (**b**) the map of the testing location at Peter Bryce Blvd, and (**c**) the OBU located in the test vehicle.

To show the effectiveness of the proposed monocone antenna in real-life V2X applications, a field test was conducted with the latest V2X communication units available on the market. To facilitate communication, MK6C C-V2X units from Cohda Wireless [31] were used for an OBU and an RSU which operates from 5.895 to 5.925 GHz. The proposed monocone antenna was connected to the OBU to transmit basic safety messages (BSMs). A commercial monopole antenna was connected to an MK6C to configure the RSU to receive the BSMs from the OBU. For testing, the RSU antenna was mounted 17 ft from the ground to mimic the average height of a traffic pole in the United States, as shown in Figure 11a. 

The field test was conducted along Bryce Lawn Road at the University of Alabama. This testing site features 700 m of a continuous line-of-sight (LOS) distance, as illustrated in Figure 11b. The RSU was placed at the starting position labeled 0 m, while the test vehicle with the OBU travels away from the RSU. The test vehicle was equipped with the OBU, as depicted in Figure 11c. The vehicle runs at 10 miles per hour and transmits BSMs at a rate of 10 Hz. Each BSM sent has a size of 191 bytes, consisting of vehicle positional data. 

**Figure 12:**
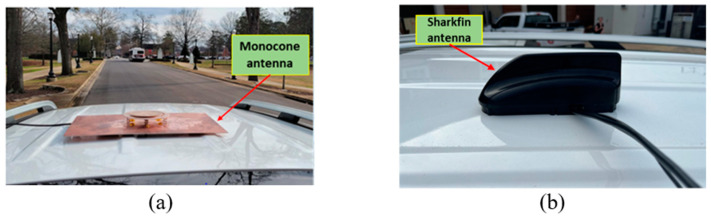
Antennas mounted on the test vehicle: (**a**) the proposed monocone antenna and (**b**) the MMXFG-5900 sharkfin-shaped antenna.

The proposed monocone antenna was placed on the roof of the vehicle and was connected to the OBU through a coaxial cable, as shown in Figure 12a. The transmission power of the OBU was set to 10 dBm in the test. For comparison, a commercial V2X sharkfin-shaped antenna (MMXFG-5900) [32,33] was tested in the same location where the proposed antennas were placed. 

The test was conducted with three different antenna configurations—the proposed monocone, a commercial antenna with single-port excitation, and the same commercial antenna with two ports excited for spatial diversity. The communication performance parameters, including the packet error rate (PER) and latency, were measured by examining the transmitted and received packets. The PER is defined as the total missed packets over the total packets sent. To examine a large enough time span to obtain accurate data on packet loss, a time window (*t*) of 20 s was applied to the PER, as shown in Equation (1).

(1) $$PER\left(t\right)=\frac{Missed\;numbers\;of\;packets(-\frac{t}{2}+1,\frac{t}{2})}{Total\;numbers\;of\;packets(-\frac{t}{2}+1,\frac{t}{2})}\;\;\left(\%\right)\;\;$$

Latency is defined as the time delay between sending and receiving a message. This is measured by the epoch time provided by each message. The equation to calculate the latency is written in Equation (2).

(2) $$\begin{array}{cc} Latency= & Timestamp\;of\;received\;packet-\\ & Timestamp\;of\;sent\;packet\left(ms\right) \end{array}$$

**Figure 13:**
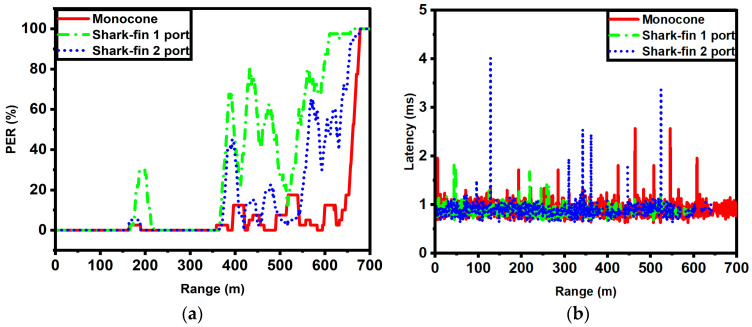
Field test results as a function of communication range for the three different antenna configurations: (**a**) packet error rate and (**b**) latency.

Five tests were conducted, and the collected data were averaged and plotted. Figure 13 shows the field test result. The ranges for the threshold of a 10% PER were found at 367 m for the one-port excited commercial antenna, 373 m for the two-port excited commercial antenna, and 396 m for the proposed antenna, respectively. The 100% PER with the proposed antenna occurred at 680 m which was much longer than that of the commercial antennas. Thus, the proposed antenna showed a lower PER with more robust connection for V2X communication relative to the widely used industry-level one- and two-port antennas.

Furthermore, the measured latency for the three different antenna configurations indicated that the proposed antenna showed fewer fluctuations in the latency values over the entire range, as shown in Figure 13b. The average latency for all three antenna conditions was similar: 1.23 ms for the commercial one-port configuration antenna, 1.12 ms for the commercial two-port configuration antenna, and lastly 1.32 ms for the proposed monocone antenna. 

Table 2 presents a comparison between the proposed antenna and the previous references. It can be seen that the proposed design offers excellent compensation between the size and the bandwidth while keeping a good gain value relative to the literature.

## 6. Conclusions

An omnidirectional monocone antenna supported with folded shorting wires was presented in this paper. The proposed design covered the V2X frequency band and beyond with stable omnidirectional radiation patterns and high efficiency from 0.75 GHz to 7.6 GHz. A fractional impedance bandwidth of 164% was achieved with a gain value as high as 10 dBi. The folded shorting wires showed significant capability to miniaturize the antenna by shifting the operating frequency to a lower band. The prototype was fabricated using 3D printing technology for commercially available plastic material and then sprayed with copper particles for appropriate coating. The antenna was fabricated and measured in the anechoic chamber and then tested in the field to validate the design performance and structure fidelity. The PER and latency results from the V2X test are very promising. 

## Figures and Tables

**Table 1:** The proposed antenna dimensions in mm or degree.

*R*_1_	*R*_2_	*R*_3_	θ _1_	θ _2_	*V_T_*	*P*_1_
55	57.6	74	35	90	2	1
*S*_1_	*V*_*m*1_	*V*_*m*2_	*V*_*m*3_	*V*_*m*4_	*V*_*m*5_	*V*_*m*6_
1.5	6	4.8	4.9	15.6	8.4	2.7
*V_TS_*	*V*_*l*1_	*V*_*l*2_	*V*_*l*3_	*V*_*l*4_	*t _top_*	*h*
2	3	4	3.7	25.4	2	26.69
*R*_*C*1_	*R*_*C*2_	*P*_*p*1_				
5.4	2.01	2.2				

**Table 2:** Comparison between the proposed antenna and previous references.

Ref	Frequency (GHz)	BW%	Gain (dBi)	$\mathrm{Dimensions}\;(\lambda_{low}^{3})$
[6]	1.6–11.7	151	3–8	0.363 × 0.363 × 0.057
[8]	2.4–7.98	107	NA	0.468 × 0.468 × NA
[9]	0.8–2.4	100	1.7–9	0.364 × 0.364 × 0.068
[18]	0.8–2.3	97	2–6	0.208 × 0.208 × 0.068
[19]	3.06–12	119	NA	0.204 × 0.204 × 0.087
[21]	1.17–30	185	2.55 @ 2 GHz	0.363 × 0.363 × 0.122
[23]	0.8–2.5	103	2–8	0.267 × 0.267 × 0.064
[24]	6.2–18	98	3.2–10.1	0.95 × 1.07 × 0.072
[26]	1.43–39.05	185.8	5.8–10.7	0.95 × 0.95 × 0.143
[34]	1.15–1.6	32	5–8.1	0.575 × 0.575 × 0.115
[35]	1.559–1.610, 2.32–2.345	N/A	2.24–5.52	0.187 × 0.187 × 0.031
[This work]	0.75–7.6	164	2–10	0.37 × 0.37 × 0.067
